# Effects of the Diamine Chain End Functionalized Liquid Butadiene Rubber as a Processing Aid on the Properties of Carbon-Black-Filled Rubber Compounds

**DOI:** 10.3390/polym14163343

**Published:** 2022-08-16

**Authors:** Sanghoon Song, Gyeongdong Yeom, Donghyuk Kim, Gyeongchan Ryu, Kiwon Hwang, Byungkyu Ahn, Haeun Choi, Hyun-Jong Paik, Sungwook Chung, Wonho Kim

**Affiliations:** 1 School of Chemical Engineering, Pusan National University, Busan 46241, Korea; 2 Department of Polymer Science and Engineering, Pusan National University, Busan 46241, Korea; 3 Hankook Tire & Technology Co., Ltd., R&D Center, 50 Yuseong-daero 935beon-gil, Yuseong-gu, Daejeon 34127, Korea

**Keywords:** liquid butadiene rubber, radical polymerization, carbon-black-filled compound, rubber compounding, truck bus radial tire tread

## Abstract

The implementation of vehicle electrification and autonomous driving technologies has recently emphasized the importance of abrasion resistance and fuel efficiency of truck bus radial (TBR) tire treads that undergo high loads and long driving times. In this study, a functionalized liquid butadiene rubber (F-LqBR) was introduced to replace the treated distillate aromatic extracted (TDAE) oil as a way to improve abrasion resistance and fuel efficiency in the TBR tire tread compound and to solve the oil migration. First, radical polymerization was used to synthesize nonfunctionalized LqBR (N-LqBR) and amino-LqBR with an amine group at the chain ends. The synthesized LqBRs were then substituted in place of TDAE oil to manufacture carbon-black-filled natural rubber (NR) compounds and to evaluate their physical properties. The results show that LqBRs improved the migration resistance and enhanced the abrasion resistance by lowering the glass transition temperature (T_g_) of the compound. In particular, amino-LqBR improved carbon black dispersion in the rubber matrix through a chemical bond between the functional group of the carbon black surface and the base rubber. In conclusion, amino-LqBR successfully served as a processing aid in a carbon black-filled NR compound while simultaneously enhancing its fuel efficiency and abrasion resistance.

## 1. Introduction

As electrification and autonomous driving technologies have been applied to commercial trucks and buses in recent years, there is increasing importance in fuel efficiency and the abrasion resistance of tires, which need to endure heavy loads and long driving times [1]. Among the various components of a tire, the tire treads, which are in direct contact with the road surface, are the most important in determining tire performance. Therefore, studies on tread compounds are essential for achieving excellent tire performance.

Unlike a passenger car radial (PCR) tire tread that uses styrene butadiene rubber (SBR), a TBR tire tread uses NR, which has excellent abrasion resistance and mechanical properties, as the base rubber [2]. As such, carbon black, which has a good affinity with NR and is excellent in reinforcing the mechanical properties, is primarily used as the filler [3,4,5]. A rubber-processing oil such as TDAE oil is partially added as a processing aid to improve the processability of the compound [6]. However, adding processing oil to the compound is not favorable for the rolling resistance and abrasion resistance of tires, resulting in a trade-off relationship between processability and physical properties [7]. In addition, the presence of processing oil causes a migration problem [8] in which the oil leaks out to the tire surface as the tire is operating for longer times, leading to a decrease in the suppleness of the tire tread and deterioration of physical properties. In particular, the migration problem in the tire treads of commercial vehicles is critical to address, as they need to drive longer distances for longer periods of time than passenger cars do.

Liquid butadiene rubber (LqBR) serves as a processing aid that is also capable of forming a crosslink with the base rubber of the compound during vulcanization, such that it experiences reduced migration compared to processing oil [9]. In addition, the glass transition temperature (T_g_) of the compound can be tuned on the basis of the microstructure of LqBR, and excellent abrasion resistance can be expected when applying LqBR with a T_g_ lower than −80 °C to lower the T_g_ of the compound itself [10]. Thus, the tire industry abounds with various studies that seek to apply LqBR as a processing aid for rubber compounds.

Kuraray Co., Ltd. (Japan) demonstrated that, via the toluene extraction of a compound to which N-LqBR is applied, the covulcanization of N-LqBR was possible, and the application of N-LqBR enhanced viscoelastic properties at low temperature and abrasion resistance [11]. SUMITOMO Rubber Industries, Ltd. (Japan) verified that oil migration associated with vehicle driving reduced the suppleness of the tread compound and deteriorated the mechanical properties. The problem was solved by reducing migration through the formation of a crosslink between N-LqBR and the base rubber, and the low-temperature performance and abrasion resistance were improved simultaneously [12]. Continental AG (Germany) applied N-LqBR to the tire tread to improve abrasion resistance without compromising the braking performance and fuel efficiency [13]. In contrast, Kitamura reported that the fuel efficiency decreases when N-LqBR is added to the compound due to the hysteresis loss that arises from the free chain ends of LqBR [14]. To limit the chain ends mobility of LqBR, the introduction of a functional group was proposed to be necessary.

In response to the need for introducing functionalized-LqBR (F-LqBR), Cray Valley Co., Ltd. (United States) and Evonik Industries AG (Germany) developed and commercialized silane-functionalized LqBR [15,16], which was subsequently applied to tire compounds in the tire industry [17,18,19]. Kim et al. reported that the ethoxysilyl group of silane-functionalized LqBR is not only bonded on the silica surface, but also forms a chemical bond with the base polymer via a crosslink reaction [20]. Therefore, it plays a similar role to that of a silane coupling agent in the compound. Moreover, owing to the limitations in its chain-end mobility, silane-functionalized LqBR significantly enhances the migration resistance and reduces hysteresis of the compound compared to N-LqBR. As a result, F-LqBR can be introduced to tire tread compounds, and applied as a processing aid for simultaneously enhancing the abrasion resistance and fuel efficiency. 

To date, most of the studies on F-LqBR have primarily focused on PCR tire treads, whereas research on TBR tire treads that undergo harsher driving conditions than those of the former is still lacking. Therefore, the purpose of this work is to introduce a compatible functional group with carbon black to LqBR and apply it as a processing aid to a carbon black-filled NR compound. First, amino-LqBR, the chain ends of which are modified with an amine group, a functional group that shows affinity to carbon black [21], is synthesized using a chain transfer agent as an iniferter (initiator + transfer agent + terminator). Amino-LqBR not only serves as a processing aid, but also forms a chemical bond between the filler and the base rubber. Furthermore, the migration problem of amino-LqBR is expected to be solved through the chemical bond. If amino-LqBR can improve the dispersion of carbon black and the mechanical properties of the compounds, it is expected to be applicable to other types of rubber compounds that use carbon black as a filler, such as conveyor belts, seals, air springs, vibration isolation devices, and hoses [22,23,24,25]. In addition, if F-LqBR is applied together with recycled carbon black, the disadvantages of recycled carbon black can be compensated; at the same time, it is a way to solve environmental problems [26,27,28].

In this study, three different compounds, each containing TDAE oil, N-LqBR, or amino-LqBR as the processing aid, were prepared, and the effect of the functional group is quantitatively investigated by analyzing the vulcanizate structure. Afterwards, the correlation between the vulcanizate structure and physical properties is explained.

## 2. Materials and Methods

### 2.1. Materials

#### 2.1.1. Polymerization

For polymerization, tetrahydrofuran (99.9%, Samchun Chemical Co., Seoul, Korea) was used as the organic solvent, and di-*tert*-butyl peroxide (DTBPO, 98%, Sigma-Aldrich Corp., Seoul, Korea) was used as the initiator. Further, 1,3-butadiene supplied by Kumho Petrochemical Co., Ltd. (Kumho Petrochemical Co., Daejeon, Korea) was used as-received, and bis(4-aminophenyl) disulfide (BAPD, Sigma-Aldrich Corp., Seoul, Korea) was used as the chain transfer agent.

#### 2.1.2. Compounding

Natural rubber (NR, Standard Vietnamese Rubber SVR-10, dirty contents 0.1 wt %, Astlett Rubber Inc., Oakville, ON, Canada) was used as the base rubber, and carbon black (N134, iodine adsorption (IA): 142 g/kg, N_2_SA adsorption specific surface area: 143 m^2^/g, OCI Company Ltd., Seoul, Korea) was used as the filler. Zinc oxide and stearic acid used as crosslinking activators were purchased from Sigma Aldrich (Seoul, Korea). N-(1,3-dimethybutyl)-N′-phenyl-phenylenediamine (6PPD, Kumho Petrochemical Co., Daejeon, Korea) and 2,2,4-trimethyl-1,2-dihydroquinoline (TMQ, Sinopec Corp., Beijing, China) were used as antioxidants. Sulfur (Daejung Chemicals & Metals Co., Siheung, Korea) was used as the crosslinking agent, and N-*tert*-butyl-2-benzothiazyl sulfenamide (TBBS, Shandong Yanggu Huatai Chemical Co., Ltd., Liaocheng, China) was used as the vulcanization accelerator. N-cyclohexylthio phthalimide (PVI, Shandong Yanggu Huatai Chemical Co., Ltd., Liaocheng, China) was used as the prevulcanization inhibitor. For processing aids, three different substances were used: TDAE oil (Kukdong Oil & Chemicals Co., Yangsan, Korea), which is commercially available, and N-LqBR and amino-LqBR, which were synthesized. Details on the synthesis method of LqBR are presented in Section 2.3.

### 2.2. Measurements

#### 2.2.1. Gel Permeation Chromatography (GPC)

The molecular weights and molecular-weight distributions were determined using gel permeation chromatography (GPC; DGU 20A 3R, Shimadzu, Kyoto, Japan) employing a solvent delivery unit, refractive index detector, and Styragel column (high temperature 6E, 10 μm, Φ 7.8 × 6300 mm; high molecular weight (HMW) 7, 15–20 μm, Φ 7.8 × 300 mm; HMW 6E, 15–20 μm, Φ 7.8 × 300 mm). The GPC calibration curve was prepared using polybutadiene standard (Kit Poly(1,4-butadiene) number average molecular weight standards, WAT035709, Waters Corp., Eschborn, Germany).

#### 2.2.2. Proton Nuclear Magnetic Resonance Spectroscopy (^1^H NMR)

The relative contents of vinyl structure in the synthesized polymer were confirmed using proton nuclear magnetic resonance spectroscopy (^1^H NMR; Varian, Unity Plus 300 spectrometer, Garden State Scientific, Morristown, NJ, USA). Deuterochloroform (CDCl_3_, Cambridge Isotope Laboratories, Inc., Andover, MA, USA) was used as a solvent, and LqBRs were dissolved at a concentration of 15 mg/mL.

#### 2.2.3. Differential Scanning Calorimetry (DSC)

The T_g_ of LqBR was measured using a differential scanning calorimeter (DSC-Q10, TA Instruments, New Castle, DE, USA). The results were obtained by heating the sample from −100 to 100 °C at a heating rate of 10 °C/min under a nitrogen atmosphere.

#### 2.2.4. Polymer Viscosity

The zero-shear viscosity of the synthesized polymers was measured using a rotational rheometer (ARES-G2, TA Instruments, New Castle, DE, USA) at a temperature of 30 °C and under shear rates in the range of 0.01–100 1/s.

#### 2.2.5. Payne Effect

To determine the degree of filler network formation in an unvulcanized compound, a strain sweep test (0.28−40.04%) was conducted at 60 °C using a rubber process analyzer (RPA 2000, Alpha Technologies, Hudson, Ohio, USA). As the applied strain was increased, the filler network was destroyed, which in turn was manifested as a decrease in the storage modulus (G’). Thus, ΔG’ (G’ at 0.28%–G’ at 40.04%) reflects the filler–filler interaction degree and describes the Payne effect [29].

#### 2.2.6. Mooney Viscosity

Mooney viscosity, which is a measure for evaluating the processibility of a compound, was characterized using a Mooney rotatory viscometer (Vluchem IND Co., Seoul, Korea). Measurements were performed in accordance with ASTM D1646 conditions by preheating for 1 min using a large rotor (diameter of 38.10 ± 0.03 mm and thickness of 5.54 ± 0.03 mm) and by operating it at 100 °C for 4 min at 2 rpm.

#### 2.2.7. Cure Characteristics

The cure characteristics of the compounds were determined using a moving die rheometer (MDR, RLR-3-rotorless rheometer, Toyoseiki, Tokyo, Japan) at 150 °C for 30 min under an angular displacement of ±1°. From this experiment, the minimal torque value (T_min_), maximal torque value (T_max_), and optimal cure time (t_90_) could be obtained. Subsequently, the optimal cure time was applied to prepare vulcanizates using a hot press at 150 °C.

#### 2.2.8. Solvent Extraction and Vulcanizate Structure

The vulcanizates were cut into dimensions of 10 (length) × 10 (width) × 2 mm (thickness) and weighed. Next, they were stored in tetrahydrofuran and n-hexane at 25 °C for two days each to remove the organic additives. Each sample was then dried at 25 °C for one day, and the mass of the sample was measured to compute the mass fraction of the extracted organic additives. The same specimen was placed in toluene at 25 °C for one day to swell and was weighed again. Lastly, on the basis of the measured mass of the sample, the crosslink density was calculated using Flory–Rehner Equation (1) shown below [30,31,32].

(1) $$\nu =\frac{1}{2M_{c}}=-\frac{\ln\left(1-V_{1}\right)+V_{1}+\chi V_{1}^{2}}{2\rho_{r}V_{0}\left(V_{1}^{\frac{1}{3}}-\frac{V_{1}}{2}\right)}$$

where *ν* is the crosslink density (mol/g), *M_C_* is the average molecular weight between crosslink points (g/mol), *V*_1_ is the volume fraction of the rubber in the swollen gel at equilibrium, *V*_0_ is the molar volume of the solvent (cm^3^/mol), *ρ_r_* is the density of the rubber sample (g/cm^3^), and *χ* is the polymer–solvent interaction parameter.

In addition, Kraus Equation (2) was used to obtain the value of *V_r_*_0_ to determine the chemical crosslink density, which is the crosslink density of an unfilled compound in the Flory–Rehner equation. Next, the degree of the filler–rubber interaction was confirmed by calculating the difference between the total crosslink density (chemical crosslink density + filler-rubber interaction) and the chemical crosslink density [33,34,35].

(2) $$\frac{V_{r0}}{V_{r}}=1-m\left(\frac{\varphi }{1-\varphi }\right)$$

where *φ* is the volume fraction of the filler, *V_r_*_0_ is the volume fraction of the unfilled rubber in the swollen gel at equilibrium, and *V_r_* is the volume fraction of the rubber in the swollen gel at equilibrium.

#### 2.2.9. Mechanical Properties

The mechanical properties (tensile strength, modulus, and elongation at break) of the vulcanizates were measured using a universal testing machine (UTM, KSU-05M-C, KSU Co., Ansan, Korea) according to ASTM D 412. Then, 100 (length) × 25 mm (width) dumbbell-shaped specimens were prepared and stretched at a rate of 500 mm/min.

#### 2.2.10. Abrasion Resistance

Cylindrical samples with diameters of 16 mm and thicknesses of 8 mm were prepared for abrasion resistance testing. After recording the initial mass of the sample, a Deutsche Industrie Normen (DIN) abrasion tester (KSU Co., Ansan, Korea) was used to subject the specimen to abrasive wear under a 5 N load at 40 rpm for 40 s. The final mass was measured to determine the amount of mass loss.

#### 2.2.11. Dynamic Viscoelastic Properties

The dynamic viscoelastic properties of the compounds were characterized using a strain-controlled rheometer (ARES-G2, TA Instruments, New Castle, DE, USA) and a dynamic mechanical thermal spectrometer (DMTS, EPLEXOR 500 N, GABO Instruments GmbH, Ahlden, Germany). First, the strain-controlled rheometer was used to measure tan ẟ by performing a temperature sweep from −60 to 70 °C at a 0.5% strain (torsion mode) and a frequency of 10 Hz. The dynamic mechanical thermal spectrometer was then used to obtain tan ẟ in the high-strain region by conducting a strain sweep from 0.5% to 10% strain (tension mode) at 60 °C and a frequency of 10 Hz.

### 2.3. Synthesis of Liquid Butadiene Rubbers

#### Radical Polymerization

**Scheme 1:**
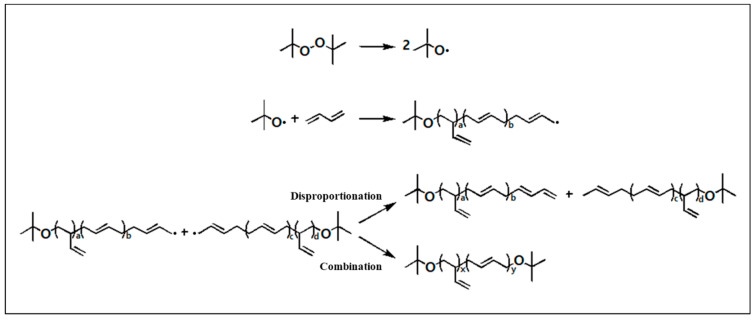
Polymerization of N-LqBR.

N-LqBR was synthesized according to the polymerization process illustrated in Scheme 1. Because no chain transfer agent was added to the synthesis of N-LqBR, a greater amount of the initiator was added to the reaction to yield a similar molecular weight to that of amino-LqBR. First, 5.849 g of DTBPO and 180 g of THF were added to a high-pressure stainless-steel stirrer reactor (1 L) at room temperature. The reactor was then purged with nitrogen five times, and 72 g of 1,3-butadiene was injected. After the injection of 1,3-butadiene, the reactor was pressurized and heated to 135 °C. When the temperature reached 135 °C, the pressure inside the reactor was approximately 50 bar, and polymerization was allowed to proceed for 4 h. Lastly, the reaction was terminated by decreasing the temperature to 15 °C.

**Scheme 2:**
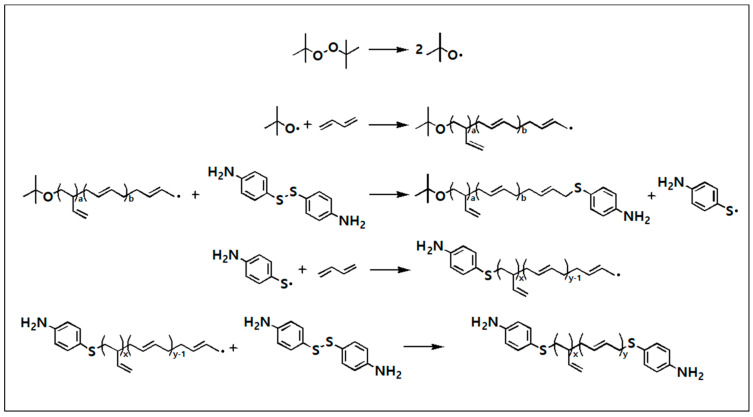
Polymerization of amino-LqBR.

The synthesis of amino-LqBR, which has amine groups at the chain ends, was conducted according to the polymerization reaction shown in Scheme 2. First, 0.487 g of DTBPO, 49.674 g of BAPD, and 180 g of THF were added to a high-pressure stainless-steel stirrer reactor (1 L) at room temperature. Next, the reactor was purged with nitrogen five times, and 180 g of 1,3-butadiene was injected. The reactor was then pressurized, and the temperature was raised to 135 °C, at which point the internal pressure was about 50 bar. Because the polymerization of amino-LqBR involved a chain transfer reaction unlike the synthesis of N-LqBR, polymerization was allowed to proceed for 8 h. Lastly, the temperature was lowered to 15 °C to terminate the reaction.

Once the reactor had reached 15 °C, unreacted 1,3-butadiene was removed through the vent line, and THF was removed using rotary evaporation. The remaining initiator and chain transfer agent were dissolved in methanol and then removed using a centrifuge. The yields of synthesized N-LqBR and amino-LqBR were 35% and 30%, respectively, and details of the polymerization formulation are presented in Table 1.

### 2.4. Manufacture of Rubber/Carbon Black Compounds and Vulcanizates

For quantitatively analyzing the vulcanizate structure of the compounds and for investigating the effect of a crosslink density on the mechanical and dynamic properties of the vulcanizates, compounds were manufactured using the formulation shown in Table 2. Compounds were manufactured using two different amounts of carbon black to divide the chemical crosslink density from the total crosslink density using the Kraus plot. In this study, the physical properties of the vulcanizates containing 55 phr of carbon black are discussed. Compounds are denoted by the respective processing aid (TDAE oil, N-LqBR, Amino-LqBR) used in the formulation.

Compounds were manufactured using an internal kneader (300 cc, MIRAESI Company, Gwangju, Korea). The fill factor was set to 0.8, and the two types of LqBRs were each added to the formulation by replacing the entire 5 phr of TDAE oil. During the first stage, kneading was started at 80 °C and was conducted for a total of 5 min 30 s. After mixing at each stage, the compound was formed into a sheet using a two-roll mill. The optimal cure time of the prepared compound at 150 °C was determined using a MDR. Thereafter, vulcanizates were prepared by vulcanization in a press at 150 °C using the optimal vulcanization time. Details of the overall procedure are presented in Table 3.

## 3. Results and Discussion

### 3.1. Synthesis of LqBR

**Figure 1:**
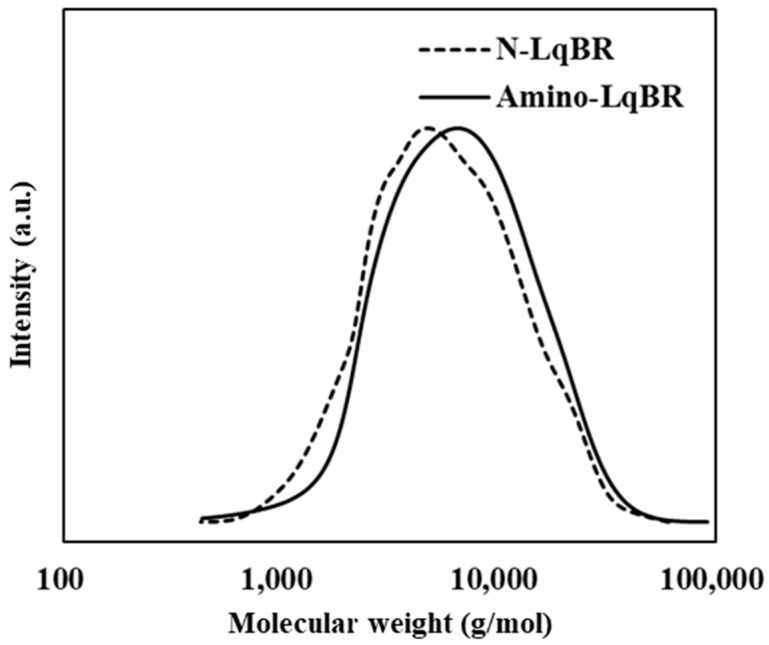
Gel permeation chromatogram of LqBRs.

**Figure 2:**
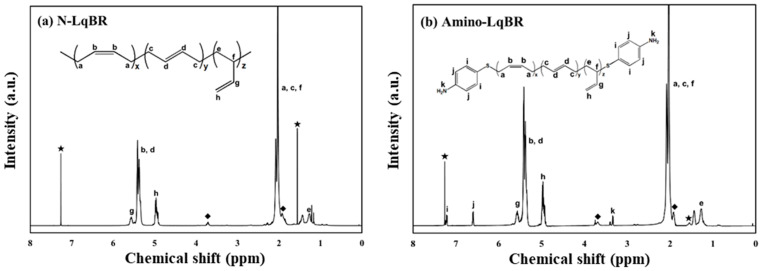
^1^H NMR spectra; (**a**) N-LqBR; (**b**) amino-LqBR (★ CDCl_3_ solvent, ◆ THF solvent).

The GPC and ^1^H NMR analysis results of the polymerized LqBRs are shown in Figure 1 and Figure 2, and Table 4. The results of the GPC measurements confirm the similarity of the molecular weight and molecular weight distribution of both N-LqBR and amino-LqBR synthesized via radical polymerization. ^1^H NMR spectra show resonance peaks corresponding to the 1,4-addition structure of butadiene at 5.3–5.5 ppm as well as those corresponding to the 1,2-addition (vinyl) structure at 5.5–5.6 and 4.8–5.0 ppm. Resonance peaks corresponding to the amino group (–NH_2_) present in the end group of phenylamine appear at 3.3–3.4 ppm, the *ortho*-position proton (–CH–C(NH_2_)–CH–) at 6.5–6.6 ppm, and the *meta*-position proton (–CH–CH–C(NH_2_)–CH–CH–) at 7.15–7.25 ppm [36].

In ^1^H NMR spectra, the area under a resonance peak for each H atom is proportional to the number of H atoms. However, various chemical shifts may occur in the resonance peak of the amine group owing to factors such as temperature, acidity, hydrogen bond strength, and solvent, which result in high variability and reduced reliability when calculating the peak area [36,37]. Therefore, the functionality, which is the number of phenylamine-containing chains with respect to the total number of LqBR chains, was determined by calculating the ratio between the proton peak area of the vinyl group and the *ortho*-position proton peak of phenylamine:

(3) $$\frac{S_{Vinyl-H}}{S_{Phenylamine-H}}=\frac{2\times \left(R_{Vinyl}\right)\times \left(M_{n}/M_{B}\right)}{n_{Phenylamine-H}\times F}$$

where *S_vinyl-H_* and *S_Phenylamine-H_* are the peak area of the hydrogen atoms of vinyl and the *ortho*-position of phenylamine, respectively; *R_Vinyl_* is the vinyl content; *M_n_* is the number-average molecular weight of LqBR; M_B_ is the molecular weight of butadiene; *n_Phenylamine_* is the hydrogen atom numbers of the *ortho*-position of phenylamine; *F* is the functionality (NH_2_/chain), i.e., “2” implies the two functionalized ends of macromolecular chains.

**Figure 3:**
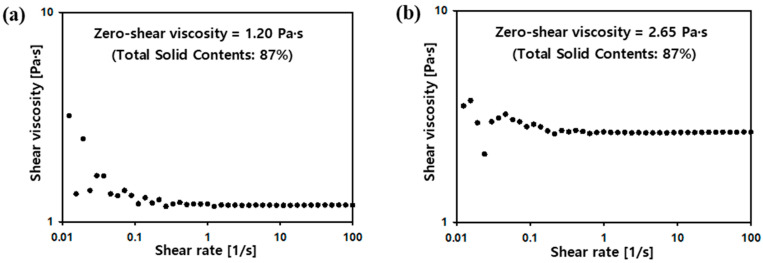
Zero-shear viscosity; (**a**) N-LqBR, (**b**) amino-LqBR.

Measurements of the zero-shear viscosity and T_g_ of the synthesized LqBR are shown in Figure 3 and Table 4. The viscosity and T_g_ of a polymer were high when the intermolecular force due to the functional groups within molecules was present [38]. In the case of amino-LqBR, the amine group at the chain ends formed an intermolecular hydrogen bond, resulting in a higher polymer viscosity than that of N-LqBR as well as higher T_g_.

### 3.2. Payne Effect

The Payne effect generally describes the extent to which a filler network is formed. The smaller the difference is in the storage modulus (ΔG′) as a result of the filler network being destroyed from increasing strain, the weaker the filler–filler interaction. On the basis of this behavior, the dispersion of fillers in the rubber matrix can be identified. In addition, in the high-strain region where the filler network was destroyed, the G’ of the compound was affected by the hydrodynamic effect, polymer network, and rubber structure [39].

**Figure 4:**
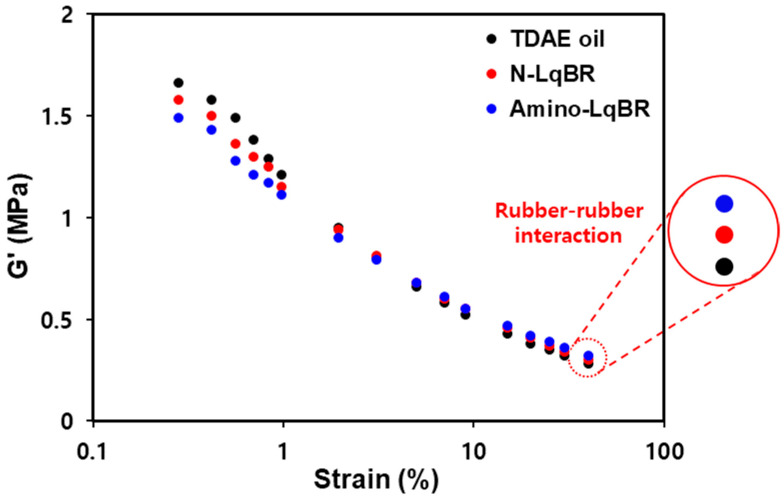
Payne effect of carbon black masterbatches.

**Figure 5:**
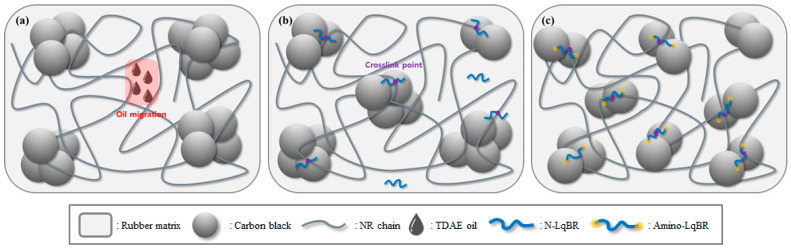
Proposed carbon black dispersion mechanism in the rubber matrix; (**a**): TDAE oil, (**b**): N-LqBR, (**c**): amino-LqBR.

The results of the measurements of the Payne effect of compounds are shown in Figure 4 and Table 5. Carbon black is a hydrophobic filler that exhibits good affinity to hydrophobic rubber. Because LqBR is a hydrophobic rubber, it was also expected to show good affinity to carbon black, and is capable of interacting with it via a Van der Waals force. As a result, the LqBR compounds showed lower Payne effect values than those of the TDAE oil compound due to the improved carbon black dispersion in the rubber matrix. In particular, the amino group at the chain ends of the amino-LqBR compound formed various chemical bonds (covalent, ionic, and hydrogen bond) with the functional group of the carbon black surface [40]. Accordingly, the dispersion of carbon black in the rubber matrix was improved, resulting in a lower Payne effect value than that of the compound prepared using N-LqBR of similar molecular weight. Figure 5 illustrates the proposed carbon black dispersion mechanism. Furthermore, the formation of chemical bonds in the amino-LqBR compound led to the highest G’ value in the high-strain region (at a 40.04% strain). This result suggests that the amino-LqBR compound may have exhibited the strongest rubber–rubber interaction among the three compounds.

### 3.3. Mooney Viscosity and Cure Characteristics of the Compounds

Table 6 shows the results of Mooney viscosity, which is a measure for assessing the processability of a compound. The obtained zero-shear viscosities of LqBRs were higher than that of TDAE oil (MW < 500). However, the Mooney viscosity values of the N-LqBR compound and TDAE oil compound were measured to be similar due to the influence of carbon black dispersion. In the case of amino-LqBR, which has a similar molecular weight to that of N-LqBR, the formation of intermolecular hydrogen bonds leads to a higher zero-shear viscosity than that of N-LqBR. As a result, the amino-LqBR compound exhibited a higher Mooney viscosity than that of the N-LqBR compound.

**Figure 6:**
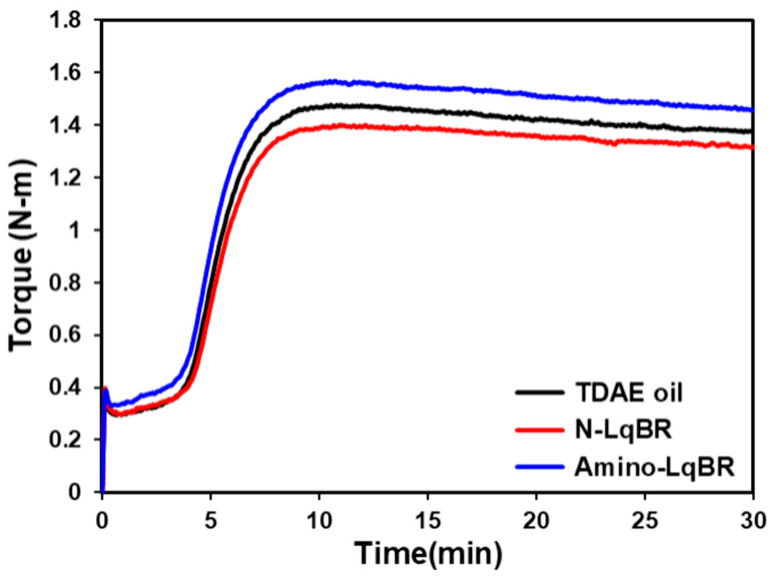
Cure characteristics of the compounds according to the applied processing aids.

The results of the cure characteristics obtained using the MDR are presented in Figure 6 and Table 6. The MDR curve shows that the Δtorque (T_max_ – T_min_) value was closely related to the crosslink density of a compound [41]. The measurement results show that the Δtorque value of the N-LqBR compound was lower than that of the TDAE oil compound. This result shows that low crosslink density was achieved, which may have been due to N-LqBR partially consuming the sulfur that was needed to crosslink the base rubber [9]. Similar to N-LqBR, amino-LqBR may also consume sulfur. However, its functional groups are not only bonded to the carbon black surface, but also form chemical bonds with the base rubber via a crosslink reaction and may enhance the filler–rubber interaction. As a result, the amino-LqBR compound showed the highest Δtorque value among the compounds.

### 3.4. Solvent Extraction and Crosslink Density

**Figure 7:**
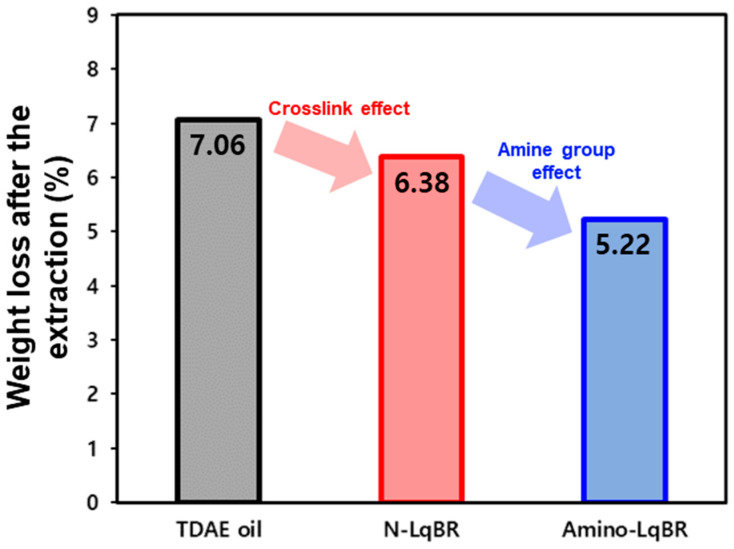
Weight loss after the extraction of the vulcanizates.

To determine the amount of organic compounds present in the vulcanizates, the organic compounds were extracted from the vulcanizates using two organic solvents. First, the oil added during compounding and low-molecular-weight chemicals were extracted with THF. Next, free LqBR that did not participate in crosslinking was extracted from the sample using *n*-hexane. The amounts of organic compounds extracted using the two organic solvents is summarized in Figure 7 and Table 7. The solvent extraction results show a very low relative standard deviation of 0.01–1.8% (error bars are not displayed).

The TDAE oil compound resulted in the highest extraction rate of organic compound at 7.06 wt% because the TDAE oil used as the processing aid acted as a plasticizer without forming a chemical bond in the vulcanizate. In contrast, N-LqBR and amino-LqBR may have been bonded to the polymer network via a crosslink with the base rubber, leading to lower extraction rates compared with those of the TDAE oil vulcanizate. In particular, amino-LqBR can be strongly bonded to the carbon black through the formation of chemical bonds between the amine group and the functional group of the carbon black surface, consequently showing a lower extraction rate than that of the N-LqBR vulcanizate. N-LqBR and amino-LqBR led to extraction rates of 77% and 37% with respect to TDAE oil assuming that the 5 phr (2.9 wt%) of TDAE oil was completely extracted, and equal amounts (4.16 wt%) of all the other additives (stearic acid, 6PPD, TMQ, etc.) were extracted.

**Figure 8:**
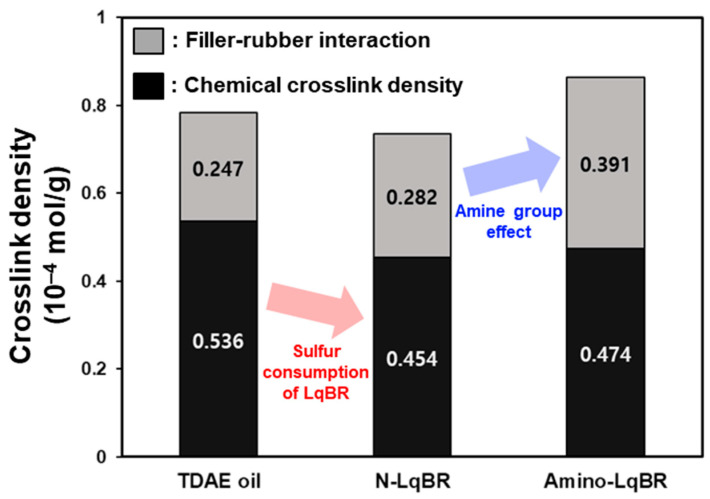
Crosslink density of the vulcanizates.

The results of vulcanizate structure analysis are shown in Figure 8 and Table 7. The total crosslink density of the manufactured vulcanizates was subdivided into chemical crosslink density and filler–rubber interaction for detailed analysis. As reflected in the relative standard deviation of 0.7–1.9%, vulcanizate structure analysis hardly showed any deviation in the results, and the error bars are not displayed. Chemical crosslink density is a numerical value indicating the crosslink density resulting from the participation of sulfur in crosslink between the rubber chains. The filler–rubber interaction represents the crosslink density formed from the bond between the filler and rubber. It was confirmed that the vulcanizates with LqBR showed a lower chemical crosslink density compared to the TDAE oil vulcanizate due to sulfur consumption of LqBR. However, unlike TDAE oil, LqBR is capable of forming a crosslink with the base rubber. Therefore, owing to the excellent affinity of LqBR to carbon black, the LqBR compounds showed a higher filler–rubber interaction than that of the TDAE oil vulcanizate. In addition, amino-LqBR forms chemical bonds with carbon black in the amino-LqBR vulcanizate, which exhibits a higher filler–rubber interaction value than the N-LqBR vulcanizate.

### 3.5. Mechanical Properties and DIN Abrasion Loss

**Figure 9:**
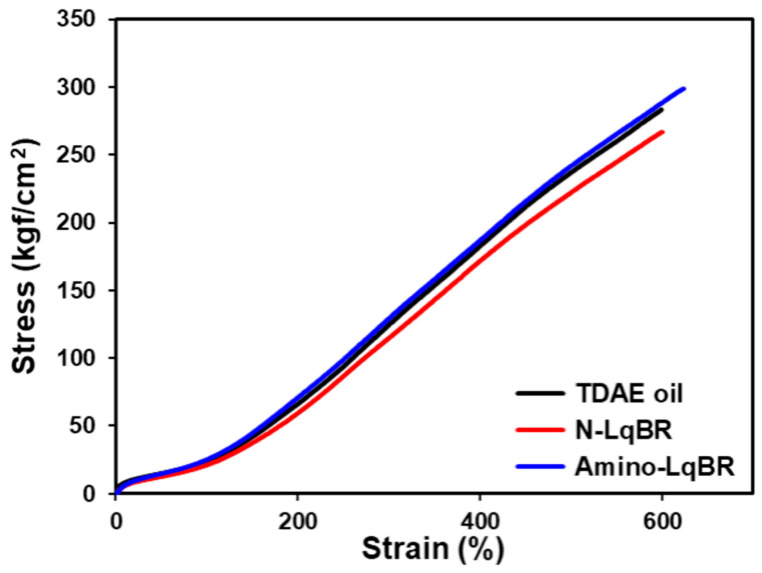
Stress–strain curves of vulcanizates.

The tensile modulus is a measure of the stiffness of rubber that is higher for a compound with a higher crosslink density [42]. As shown in Figure 9 and Table 8, the N-LqBR compound resulted in the lowest M_100_ (modulus at 100% elongation) and M_300_ (modulus at 300% elongation). This is because it has low chemical crosslink density owing to sulfur consumption and a lower total crosslink density than that of the TDAE oil compound. However, owing to the enhanced filler–rubber interaction arising from the chemical bonds, the amino-LqBR compound exhibited the highest total crosslink density, which leads to the highest M_100_ and M_300_ values.

The results of DIN abrasion tests show that the stronger the filler–rubber interaction in the compound and the lower the T_g_ of the polymer were, the more resistant the material was to abrasion loss [43,44]. The manufactured compounds were prepared using the same base rubber, but were added with different processing aids varying in T_g_. Therefore, the TDAE oil compound, which showed the weakest filler–rubber interaction and contained the processing aid with the highest T_g_ of −44 to −50 °C [45] exhibited the worst abrasion resistance among the three vulcanizates. The T_g_ of amino-LqBR (T_g_: −81 °C) was higher than that of N-LqBR (T_g_: −88 °C), but the compound prepared using amino-LqBR showed stronger filler–rubber interaction and the best abrasion resistance among the three vulcanizates.

### 3.6. Dynamic Viscoelastic Properties

When a tire rotates under the load of a vehicle, all parts of the tire undergo energy loss through repeated deformation and recovery. This phenomenon is known as hysteresis loss. When the dynamic viscoelastic properties are analyzed in the low-temperature region where the motion of the filler is restricted, the micro-Brownian motion of the polymer is the main cause of energy dissipation in the network. In this regard, a higher volume fraction of the polymer, near its T_g_, increases the hysteresis loss of the compound [46]. Tan δ at 60 °C is a measure related to the hysteresis loss that occurs during the destruction and reformation of the filler network. Its higher value is more unfavorable for the rolling resistance of the compound [2,46]. In addition, tan δ at 60 °C is lower when the filler–rubber interaction is stronger, and the filler dispersion is more uniform [9,46].

**Figure 10:**
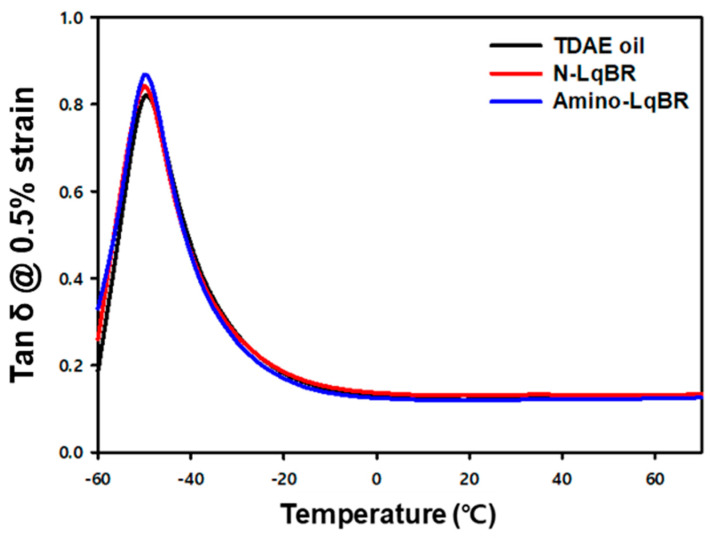
Tan δ curves of vulcanizates in the temperature sweep test.

Tan δ with respect to temperature was measured using a strain-controlled rheometer under 0.5% strain, as shown in Figure 10 and Table 9. The measurement results reveal that the compounds containing LqBR had higher tan δ at T_g_ values than those of the TDAE oil compound. This is because, as confirmed in the Payne effect results, the effective filler volume fraction decreased as the dispersion of carbon black in the compound was improved, and thus the volume fraction of the polymer was relatively increased. Moreover, the N-LqBR compound showed higher tan δ at 60 °C values than those of the TDAE oil compound because of the hysteresis loss resulting from the free chain ends of N-LqBR. In contrast, chain ends of the amino-LqBR are chemically bonded to carbon black, such that the free-chain end effect is absent in the amino-LqBR compound, which shows similar tan δ at 60 °C values to those of the TDAE oil compound.

**Figure 11:**
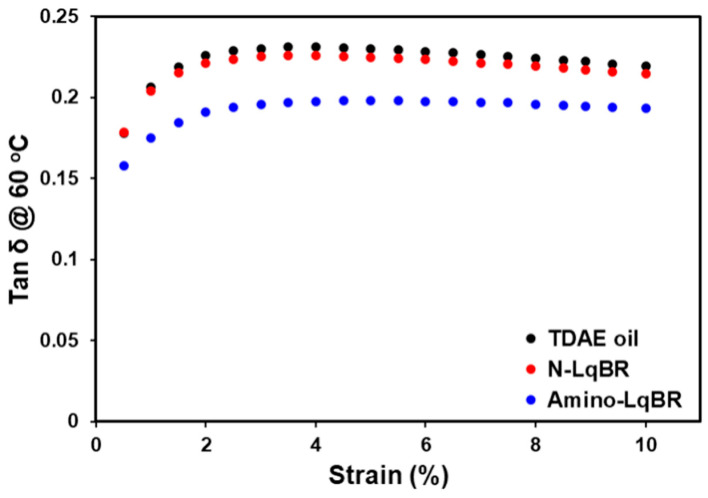
Tan δ curves of vulcanizates in the strain sweep test.

Figure 11 and Table 9 show the results of the measurements on tan δ obtained from strain sweep tests at 60 °C using the DMTS. Unlike in the low-strain region, the filler network showed structural destruction in the high-strain region (applied strain > 5%), and this behavior is reflected as a more pronounced effect of hysteresis due to the structural destruction [47,48]. Thus, the better the dispersion is, the less the filler network is formed, so a low tan δ at 60 °C under high strain can be expected. The LqBR compounds showed better dispersion of carbon black than the TDAE oil compound, resulting in less of a formation of filler networks. Consequently, the LqBR compounds exhibited low tan δ at 60 °C under high strains because of low hysteresis loss from the filler network destruction. In particular, the amino-LqBR compound showed the lowest tan δ at 60 °C in the high-strain region owing to its excellent carbon black dispersion and strongest filler–rubber interaction.

## 4. Conclusions

The test results show that LqBR not only serves as a processing aid, but also forms a crosslink with the base rubber to suppress migration. In particular, amino-LqBR, which contains a functional group and forms various chemical bonds with the carbon black surface, exhibited superior migration resistance compared to N-LqBR. Additionally, LqBR compounds showed excellent filler dispersion compared to the TDAE oil compound owing to the good affinity between LqBR and carbon black. Furthermore, the amino-LqBR compound resulted in the lowest Payne effect value because of the formation of chemical bonds.

**Figure 12:**
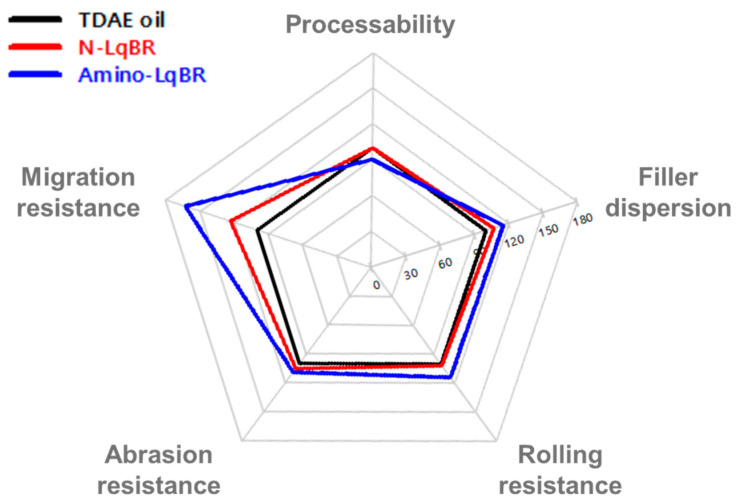
Performance of compounds with different processing aids applied.

The LqBR compounds showed a lower chemical crosslink density than that of the TDAE oil compound owing to the presence of a double bond in the LqBR chain resulting in sulfur consumption. However, the amino-LqBR compound exhibited strong filler–rubber interaction arising from the functional group and resulted in a higher total crosslink density than that of the TDAE oil compound. In addition, the vulcanizate structure of the compounds and their physical properties demonstrated a strong correlation. The tensile modulus showed a strong dependence on the total crosslink density and was the highest in the amino-LqBR compound. The abrasion resistance of the N-LqBR compound was superior to that of the TDAE oil compound owing to the low T_g_ of the polymer and the strong filler–rubber interaction. Particularly, the amino-LqBR compound showed the best abrasion resistance originating from its strong filler–rubber interaction. The dynamic viscoelastic properties reveal differences in the tan δ at 60 °C behavior between the low-strain and high-strain regions. In the low-strain region, hysteresis loss originating from the free chain end of a polymer was the main factor governing tan δ at 60 °C. In the high-strain region, the degree of filler network destruction and extent of filler–rubber interaction formation were the main factors of hysteresis loss. As such, the amino-LqBR compound in which chemical bonds with the carbon black surface were formed showed the lowest tan δ at 60 °C in the high-strain region because of its improved carbon black dispersion and excellent filler–rubber interaction. The performance of compounds with different processing aids applied is shown in Figure 12. 

In summary, when TDAE oil was replaced with amino-LqBR and applied as a processing aid for TBR tire tread, the Mooney viscosity of the compound slightly increased due to the high viscosity of the polymer. However, this improved migration resistance and filler dispersion, and increased filler-rubber interaction, resulting in the excellent abrasion resistance and fuel efficiency of compound. This study confirmed the effect of introducing F-LqBR as a processing aid in a carbon black-filled NR compound. In addition, the increase in filler–rubber interaction from the incorporation of a functional group was quantified using vulcanizate structure analysis, and the relationship between the vulcanizate structure and the physical properties was explored. Unlike previous F-LqBR studies that focused on PCR tire tread compound, this study derived new research results on the TBR tire tread compound and demonstrated the superiority of F-LqBR, which has a new structure. The results presented in this study verified the excellence of amino-LqBR, which can simultaneously improve fuel efficiency and abrasion resistance in a TBR tire tread compound with high-load and long-time driving conditions, and prevent deterioration of physical properties due to changes over time. Through the results of this study, we confirmed the applicability of F-LqBR to improve the physical properties of TBR tire tread compounds.

## Figures and Tables

**Table 1:** Formulation for the polymerization of LqBRs (Unit: g, mol).

	Organic Compounds	N-LqBR		Amino-LqBR	
		g	mol	g	mol
Initiator	DTBPO	5.849	0.04	0.487	0.0033
Chain transfer agent	BAPD	0	0	49.674	0.20
Monomer	1,3-butadiene	72	1.33	180	3.33
Solvent	THF	180	2.50	180	2.50

**Table 2:** Formulation of compounds (unit: phr).

Sample Code	TDAE Oil	N-LqBR	Amino-LqBR
NR	100	100	100
Carbon black (N134)	35/55	35/55	35/55
TDAE oil	5	-	-
N-LqBR	-	5	-
Amino-LqBR	-	-	5
Zinc oxide	4	4	4
Stearic acid	3	3	3
6PPD	2	2	2
TMQ	1	1	1
Sulfur	1.3	1.3	1.3
TBBS	1.1	1.1	1.1
PVI	0.3	0.3	0.3

Notes: phr, parts per hundred rubber.

**Table 3:** Mixing procedures.

	Time, min	RPM	Action
Carbon black masterbatch (CMB) mixing	00:00–01:30	20	NR mastication (initial temperature: 80 °C)
	01:30–02:30	40	Add carbon black ^1^/_2_, processing aid ^1^/_2_
	02:30–03:30	40	Add carbon black ^1^/_2_, processing aid ^1^/_2_
	03:30–05:30	50	Add ZnO, stearic acid, 6PPD, TMQ and dump (dump temperature: 135–140 °C)
Final masterbatch (FMB) mixing	00:00–00:30	20	Add CMB (initial temperature: 60 °C)
	00:30–02:30	40	Add sulfur, TBBS, PVI and dump (dump temperature: 80–90 °C)

**Table 4:** Characteristics of LqBRs.

Polymer	Unit	N-LqBR	Amino-LqBR
Sample M_n_	g/mol	3900	4300
Polydispersity index (PDI)	-	1.66	1.71
Vinyl content (% in BD)	-	20	20
T_g_	°C	−88	−81
Functionality (NH_2_/chain)	-	0	2.24

**Table 5:** G′ values according to the processing aids applied to the compounds.

Compound	Unit	TDAE Oil	N-LqBR	Amino-LqBR
G′ (at a 0.28% strain)	MPa	1.66	1.58	1.49
G′ (at a 40.04% strain)	MPa	0.28	0.30	0.32
△G′ (at a 0.28–40.04% strain)	MPa	1.38	1.28	1.17

**Table 6:** Cure characteristics and Mooney viscosity of the compounds.

Compound	Unit	TDAE Oil	N-LqBR	Amino-LqBR
Mooney viscosity (ML_1+4_ at 100 °C, FMB)	MU	73	73	80
T_min_	N-m	0.30	0.30	0.33
T_max_	N-m	1.48	1.40	1.57
ΔTorque	N-m	1.18	1.10	1.24
t_10_	min:s	3:53	4:00	3:40
t_90_	min:s	7:17	7:27	7:11

**Table 7:** Weight loss after the extraction and crosslink density of the vulcanizates.

Compound	Unit	TDAE Oil	N-LqBR	Amino-LqBR
Weight loss after the extraction	%	7.06	6.38	5.22
Weight loss after the extraction in 5 phr of oil and LqBR	%	100	77	37
Total crosslink density	10^−4^ mol/g	0.783	0.736	0.865
Chemical crosslink density	10^−4^ mol/g	0.536	0.454	0.474
Filler–rubber interaction	10^−4^ mol/g	0.247	0.282	0.391

**Table 8:** Mechanical properties and abrasion resistance of vulcanizates.

Compound	Unit	TDAE Oil	N-LqBR	Amino-LqBR
M_100_	kgf/cm^2^	24	21	25
M_300_	kgf/cm^2^	124	115	129
Elongation at break	%	599	600	624
Tensile strength	kgf/cm^2^	283	267	299
DIN abrasion loss	mg	88	84	80

**Table 9:** Viscoelastic properties of vulcanizates.

Compound	Unit	TDAE Oil	N-LqBR	Amino-LqBR
Number of free chain ends	-	N/A	2	0
T_g_	°C	−49.2	−50.1	−49.7
Tan δ at T_g_	-	0.822	0.848	0.867
Tan δ at 60 °C (0.5% strain, temperature sweep)	-	0.124	0.132	0.124
Tan δ at 60 °C (5% strain, strain sweep)	-	0.230	0.225	0.198

## Data Availability

Data presented in this study are available upon request from the corresponding author.

## References

[1] Rodgers B. (2020). Tire Engineering: An Introduction.

[2] Han S., Kim W.S., Mun D.Y., Ahn B., Kim W. (2020). Effect of coupling agents on the vulcanizate structure of carbon black filled natural rubber. Compos. Interfaces.

[3] Xu Z., Song Y., Zheng Q. (2019). Payne effect of carbon black filled natural rubber compounds and their carbon black gels. Polymer.

[4] Farida E., Bukit N., Ginting E.M., Bukit B.F. (2019). The effect of carbon black composition in natural rubber compound. Case Stud. Therm. Eng..

[5] Omnès B., Thuillier S., Pilvin P., Grohens Y., Gillet S. (2008). Effective properties of carbon black filled natural rubber: Experiments and modeling. Compos. A Appl. Sci. Manuf..

[6] Ezzoddin S., Abbasian A., Aman-Alikhani M., Ganjali S.T. (2013). The influence of non-carcinogenic petroleum-based process oils on tire compounds’ performance. Iran. Polym. J..

[7] Sökmen S., Oßwald K., Reincke K., Ilisch S. (2021). Influence of treated distillate aromatic extract (TDAE) content and addition time on rubber-filler interactions in silica filled SBR/BR blends. Polymers.

[8] Corman B.G., Deviney M.L., Whittington L.E. (1970). The migration of extender oil in natural and synthetic rubber. IV. Effect of saturates geometry and carbon black type on diffusion rates. Rubber Chem. Technol..

[9] Kim D., Ahn B., Kim K., Lee J., Kim I.J., Kim W. (2021). Effects of molecular weight of functionalized liquid butadiene rubber as a processing aid on the properties of SSBR/silica compounds. Polymers.

[10] Iz M., Kim D., Hwang K., Kim W., Ryu G., Song S., Kim W. (2020). The effects of liquid butadiene rubber and resins as processing aids on the physical properties of SSBR/silica compounds. Elastom. Compos..

[11] Gruendken M. Liquid rubber for safer and faster tires. Proceedings of the Tire Technology EXPO 2018.

[12] Ikeda K. Bio liquid polymer for winter tires. Proceedings of the Tire Technology EXPO 2018.

[13] Sierra V.P., Wagemann J., Van De Pol C., Kendziorra N., Herzog K., Recker C., Mueller N. (2015). Rubber Blend with Improved Rolling Resistance Behavior. U.S. Patent.

[14] Kitamura T., Lawson D.F., Morita K., Ozawa Y. (1995). Anionic Polymerization Initiators and Reduced Hysteresis Products Therefrom. U.S. Patent.

[15] Cray Valley, Technical Data Sheet, Recon. http://www.crayvalley.com/docs/tds/ricon-603-.pdf?sfvrsn=2.

[16] Evonik Less Fuel and Lower CO2 Emissions with POLYVEST ST Tires. https://coatings.evonik.com/en/less-fuel-andlower-CO2-emissions-with-polyvest-st-tires-100233.html.

[17] Herpich R., Fruh T., Heiliger L., Schilling K. (2003). Silica Gel-Containing Rubber Compounds with Organosilicon Compounds as Compounding Agent. U.S. Patent.

[18] Takuya H., Tochiro M. (2003). Tire Tread Rubber Composition. J.P. Patent.

[19] Satoyuki S., Chikashi Y. (2005). Rubber Composition Containing Compound Having Organosilicon Function Group through Urethane Bond at Terminal. J.P. Patent.

[20] Kim D., Yeom G., Joo H., Ahn B., Paik H.J., Jeon H., Kim W. (2021). Effect of the functional group position in functionalized liquid butadiene rubbers used as processing aids on the properties of silica-filled rubber compounds. Polymers.

[21] Iyama H., Ozturk O., Sumitomo Chemical Co., Ltd. (2016). Performance improvement of natural rubber/carbon black composites by novel coupling agents. Sumitomo Kagaku.

[22] He S.J., Wang Y.Q., Xi M.M., Lin J., Xue Y., Zhang L.Q. (2013). Prevention of oxide aging acceleration by nano-dispersed clay in styrene-butadiene rubber matrix. Polym. Degrad. Stab..

[23] Jansinak S., Markpin T., Wimolmala E., Mahathanabodee S., Sombatsompop N. (2018). Tribological properties of carbon nanotube as co-reinforcing additive in carbon black/acrylonitrile butadiene rubber composites for hydraulic seal applications. J. Reinf. Plast. Compos..

[24] Seo J., Kim D., Park H., Seo K. (2014). Research on CR/Nylon 6 cord rubber sleeve of rubber air spring. Elastomers Compos..

[25] Yurovska I., Gaudet G. (2008). Using furnace blacks in hose compounds. Rubber & Plastics News.

[26] Dwivedi C., Manjare S., Rajan S.K. (2020). Recycling of waste tire by pyrolysis to recover carbon black: Alternative & environment-friendly reinforcing filler for natural rubber compounds. Compos. Part B Eng..

[27] Ismawi D.H.A., Zaeimoedin T.Z., Saad C.S.M. Recovered carbon black (rCB) from waste tyres: Effect on mechanical properties of rubber compound. Proceedings of the Conference Malaysian Science and Technology Congress (MSTC 2010).

[28] Moulin L., Da Silva S., Bounaceur A., Herblot M., Soudais Y. (2017). Assessment of recovered carbon black obtained by waste tires steam water thermolysis: An industrial application. Waste Biomass Valoriz..

[29] Ramier J., Gauthier C., Chazeau L., Stelandre L., Guy L. (2007). Payne effect in silica-filled styrene–butadiene rubber: Influence of surface treatment. J. Polym. Sci. B Polym. Phys..

[30] Lee J.Y., Park N., Lim S., Ahn B., Kim W., Moon H., Paik H.J., Kim W. (2017). Influence of the silanes on the crosslink density and crosslink structure of silica-filled solution styrene butadiene rubber compounds. Compos. Interfaces.

[31] Boonstra B.B., Taylor G.L. (1965). Swelling of filled rubber vulcanizates. Rubber Chem. Technol..

[32] Verbruggen M.A.L., Van Der Does L., Noordermeer J.W., Van Duin M., Manuel H.J. (1999). Mechanisms involved in the recycling of NR and EPDM. Rubber Chem. Technol..

[33] Ahn B., Kim D., Kim K., Kim I.J., Kim H.J., Kang C.H., Lee J.Y., Kim W. (2019). Effect of the functional group of silanes on the modification of silica surface and the physical properties of solution styrene-butadiene rubber/silica composites. Compos. Interfaces.

[34] Lee J.Y., Ahn B., Kim W., Moon H., Paik H.J., Kim W. (2017). The effect of accelerator contents on the vulcanizate structures of SSBR/silica vulcanizates. Compos. Interfaces.

[35] Han S., Gu B., Kim S., Kim S., Mun D., Morita K., Kim D., Kim W. (2020). Effect of sulfur variation on the vulcanizate structure of silica-filled styrene-butadiene rubber compounds with a sulfide–silane coupling agent. Polymers.

[36] Shefer A., Grodzinsky A.J., Prime K.L., Busnel J.P. (1993). Free-radical telomerization of tert-butyl acrylate in the presence of bis (4-aminophenyl) disulfide as a useful route to amino-terminated telomers of poly (acrylic acid). Macromolecule.

[37] Pavia D.L., Lampman G.M., Kriz G.S., Vyvyan J.A. (2014). Introduction to Spectroscopy.

[38] Rothfuss N.E., Petters M.D. (2017). Influence of functional groups on the viscosity of organic aerosol. Environ. Sci. Technol..

[39] Jayalakshmy M.S., Mishra R.K. (2019). Applications of carbon-based nanofiller-incorporated rubber composites in the fields of tire engineering, flexible electronics and EMI shielding. Carbon-Based Nanofillers and Their Rubber Nanocomposites.

[40] Wang X., Wu L., Xiao T., Yu H., Li H., Yang J. (2022). Preparation and application of carbon black-filled rubber composite modified with a multi-functional silane coupling agent. Int. Polym. Process..

[41] Choi S.S., Kim I.S., Woo C.S. (2007). Influence of TESPT content on crosslink types and rheological behaviors of natural rubber compounds reinforced with silica. J. Appl. Polym. Sci..

[42] Zhao F., Bi W., Zhao S. (2011). Influence of crosslink density on mechanical properties of natural rubber vulcanizates. J. Macromol. Sci. B.

[43] Ryu G., Kim D., Song S., Hwang K., Kim W. (2021). Effect of the epoxide contents of liquid isoprene rubber as a processing aid on the properties of silica-filled natural rubber compounds. Polymers.

[44] Halasa A.F., Prentis J., Hsu B., Jasiunas C. (2005). High vinyl high styrene solution SBR. Polymer.

[45] Isitman N.A.H., Thielen G.M.V. (2018). Rubber Composition and Pneumatic Tire. E.U. Patent.

[46] Wang M.J. (1998). Effect of polymer-filler and filler-filler interactions on dynamic properties of filled vulcanizates. Rubber Chem. Technol..

[47] Wang M.J., Kutsovsky Y., Zhang P., Murphy L.J., Laube S., Mahmud K. (2002). New generation carbon-silica dual phase filler part I. Characterization and application to passenger tire. Rubber Chem. Technol..

[48] Wang M.J., Zhang P., Mahmud K. (2001). Carbon—silica dual phase filler, a new generation reinforcing agent for rubber: Part IX. Application to truck tire tread compound. Rubber Chem. Technol..

